# Supplementary material for: Cytotoxic Effects of Combinative ZnPcS4 Photosensitizer Photodynamic Therapy (PDT) and Cannabidiol (CBD) on a Cervical Cancer Cell Line
Source: Int J Mol Sci. 2023 Mar 24;24(7):6151. doi: 10.3390/ijms24076151 (PMC10094677; doi:10.3390/ijms24076151)
Supplement: Supplementary file 1 [file ijms-24-06151-s001.zip › ijms-2168337-supplementary.pdf]

Supplementary

Cytotoxic Effects of Combinative $ZnPcS_4$ Photosensitizer Photodynamic Therapy (PDT) and Cannabidiol (CBD) on a Cervical Cancer Cell Line

1. *Flow Cytometry Cell Death Pathway Analysis of $ZnPcS_4$ PS and CBD PDT / Irradiation Combinative Assays*

The Annexin V-FITC/PI cell death detection kit (BD Pharmingen™ Scientific: BD/556570) was used for detection and quantitation of cells undergoing early or late apoptosis, cells dying from necrosis cells, or cells remaining viable within combinative $ZnPcS_4$ PS and CBD PDT response assays 24 h post irradiation, using a BD Accuri™ C6 flow cytometer. All experimentation instructions and controls for gating were included as per the manufacturers' protocol recommendations: https://www.bdbiosciences.com/en-eu/products/reagents/flow-cytometry-reagents/research-reagents/panels-multicolor-cocktails-ruo/fitc-annexin-v-apoptosis-detection-kit-ii.556570.

Briefly, cellular suspensions in microcentrifuge tubes were centrifuged at 2200 rpm for 4 minutes at 20°C. Their supernatants were and cell pellets were re-suspended in 1x (v/v) Binding Buffer to a 1×10^5 cells/ml. The samples were centrifuged and 100 µl of each cellular suspension was stained with 5 µl of Annexin V-FITC solution and 5 µl of Reconstituted Propidium Iodide Staining Solution. Before the acquisition, 400 µL of Binding Buffer 1X was added. For each sample, 20,000 events were acquired using a BD Accuri™ C6 flow cytometer, with a 488 nm solid-state laser (40 mW) and optimal photomultiplier (PMT) voltages were established for each channel. The matching BD Accuri C6 Plus Annexin V-FITC/PI software kit template was used for data acquisition. Debris and doublets were excluded from the analysis. Early and late apoptotic cells were identified for their positivity to Annexin V, and necrotic cells were identified for their positivity to PI, as well as live cells being identified as per non stain controls as shown in the FACS gating strategy represented in *below*. FlowJo v 10.8.1 Software (BD Biosciences) was used for data analysis. To obtain comparable results, prior to sample analyses all quality control procedures and calibrations were performed on the flow cytometer using BD Pharmingen™ Scientific Flow Maintenance and Flow Starter Kits, with tracking beads.

According to the manufacturer's protocol referenced above, the membrane phospholipid known as phosphatidylserine is typically located in the inner surface of the cell membrane. During apoptosis, phosphatidylserine becomes translocated to the outer surface of the plasma membrane, exposing it to the external environment. Annexin V is a protein that has high affinity for exposed phosphatidylserine in the presence of calcium. To analyze the binding of the Annexin V to the phosphatidylserine, Annexin V conjugated to FITC was used to detect the apoptotic fluorescent staining of cells. Staining with Annexin V-FITC precedes the loss of membrane integrity, which accompanies the latest stages of cell death resulting from apoptotic processes. Therefore, staining with Annexin V was used in conjunction with a propidium iodide (PI), a pivotal and specific dye use to identify necrotic cells. This is because healthy cells with integral membranes exclude PI, while dead and damaged cells take up PI, due to their disrupted membranes. Hence, when interpreting flow cytometry scatter plot quadrant results after gating, viable cells with intact cell membranes are both Annexin V and PI stain negative, whereas cells undergoing early apoptosis are Annexin V stain positive and

PI stain negative, and cells that are in late apoptosis are both Annexin V and PI stain positive, while necrotic cells are only PI stain positive *(Figure S1)*.

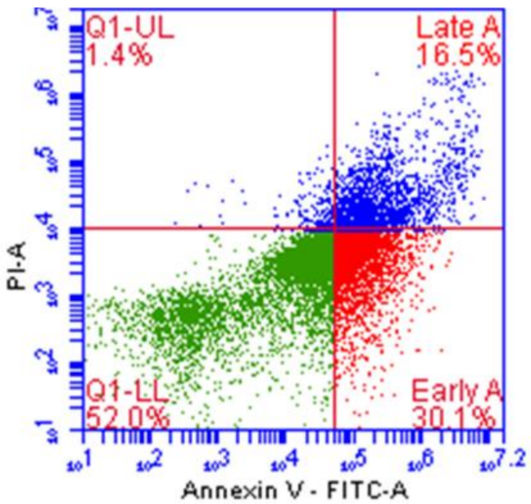

Figure S1. Flow cytometry FACS scatter plot and gating strategy; cells negative for both Annexin V-FITC and PI are viable (lower left quadrant), whereas cells positive for PI indicate cells are undergoing necrosis (upper left quadrant), cells positive for both Annexin V-FITC and PI represent late apoptosis (upper right quadrant) and cells positive for Annexin V-FITC and negative for PI are in early apoptosis (lower right quadrant).

The maximum absorption of Annexin V-FITC is 492 nm, and the maximum emission is 520 nm, whereas the maximum absorption for PI is 370 nm with an emission range of 560 to 680 nm. Thus, staining with both Annexin V-FITC and PI enables the detection and differentiation of viable cells, cells in the early apoptotic or late apoptotic phases, as well as necrotic phases of cell death.

Various negative and positive control groups consisting of either HeLa or WS1 cells in cellular suspension were used to set up and stained for flow cytometry compensation and various quadrant gating restrictions for study reliability, according to the flow cytometry kit manufacturer's protocols *(Table S1)*.

Table S1. Description of various volumes and concentrations which flow cytometry control groups of HeLa and WS1 cell culture suspensions received for $ZnPcS_4$ PS and CBD PDT combinative response assays.

Plate No.	HeLa or WS1 Culture Plate Contents	Culture Plate Contents	Flow Cytometry Staining to Cellular Suspensions	Group Type
1	Cells only	3 ml media	No staining applied	Negative Control
2	Cells only	3 ml media	5 µl of Annexin V-FITC staining solution only	Positive Annexin Control
3	Cells only	3 ml media	5 µl of PI staining solution only	Positive PI Control
4	Cells only	3 ml media	5 µl of Annexin V-FITC and 5 µl of PI staining solution	Positive Control Viable Cells
5	Cells only	1 ml media + of 3 % (v/v) Formaldehyde Solution	5 µl of Annexin V-FITC and 5 µl of PI staining solution	Positive Control Apoptosis
6	Cells only	1 ml media + 500 µl of concentrated hydrogen peroxide	5 µl of Annexin V-FITC and 5 µl of PI staining solution	Positive Control Necrosis

An apoptosis positive control *(Table S1, plate no. 5)* was included in this study that consisted of HeLa or WS1 cells cultured cells only and they were stimulated to undergo apoptosis by adding 1 ml of 3.7 % (v/v) formaldehyde solution in 0.01 M PBS to their cell culture plate which contained only 1 ml of complete cell culture medium. The culture plates were then incubated at 37°C for 30 minutes to induce cellular apoptosis. Thereafter the apoptotic cellular suspension was dispensed into a microcentrifuge tube and centrifuged at 2200 rpm for 4 minutes at 20°C, to obtain a pellet for cellular flow cytometry suspension staining and analysis.

A necrosis positive control *(Table S1, plate no. 6)* of either HeLa or WS1 cultured cells was established in this study by adding 500 µl of hydrogen peroxide solution, 30 % (w/w) in water to a culture plate containing 1 ml of complete cell culture medium. The culture plates were then incubated at 37°C for 20 minutes for necrosis to be induced. After this the necrotic cellular suspension was dispensed into a microcentrifuge tube and centrifuged at 2,200 rpm for 4 minutes at 20°C, to obtain a pellet for cellular flow cytometry suspension staining and analysis.

With reference to *Table S1 and Figure S1*, for the overall percentage of apoptotic (early-stage and late-stage), necrotic and viable cells to be calculated within the various experimental and control groups samples, positive control groups *(plate nos. 4, 5 and 6)* were used to define the basal level of viable, total apoptotic and necrotic cells within experimental set up. The total percentage of viable cells within the experimental set up was calculated from the lower left quadrants of statistically analyzed FACS scatter grams *(Figure S1)*, by subtracting the percentage values of viable cells in sample experimental and control groups, from the percentage value of viable cells obtained for experimental control group *(plate no. 4)*. The total percentage of early-stage apoptotic cells within the experimental set up was calculated from the lower right quadrants of statistically analyzed FACS scatter grams *(Figure S1)*, by subtracting the percentage values of early-stage apoptotic cells in sample experimental and control groups, from the percentage value of apoptotic cells obtained for positive control group *(plate no. 5)*. The total percentage of late-stage apoptotic cells within the experimental set up was calculated from the upper right quadrants of statistically analyzed FACS scatter grams *(Figure S1)*, by subtracting the percentage values of late-stage apoptotic cells experimental and control groups samples, from the percentage value of apoptotic cells obtained for positive control group *(plate no. 5)*. The total percentage of necrotic cells within the experimental set up was calculated from the upper left quadrants of statistically analyzed FACS scatter grams *(Figure S1)*, by subtracting the percentage values of necrotic cells in experimental and control groups samples, from the percentage value of necrotic cells obtained for positive control group *(plate no. 6)*.

The type of cell death HeLa or WS1 cells underwent post-irradiation for the various control and experimental sample groups of combinative $ZnPcS_4$ PS and CBD PDT response assays was measured in triplicate and the differences were graphically presented to determine the combinative cellular death induction effects this treatment had on HeLa, as well as determine if there were any unwanted side effects on normal cells.
